# Project-based engineering learning in college: associations with self-efficacy, effort regulation, interest, skills, and performance

**DOI:** 10.1007/s43545-021-00286-4

**Published:** 2021-12-05

**Authors:** Liang Li Wu, Christian Fischer, Fernando Rodriguez, Gregory N. Washington, Mark Warschauer

**Affiliations:** 1 Henry Samueli School of Engineering, University of California, Irvine, Irvine, USA; 2 Hector Research Institute of Education Sciences and Psychology, University of Tübingen, Tübingen, Germany; 3 School of Education, University of California, Irvine, Irvine, USA; 4 College of Engineering and Computing, George Mason University, Fairfax, VA USA

**Keywords:** Project-based learning, Engineering education, Hybrid learning, Motivation

## Abstract

This quantitative study examined student participation in an introductory project-based engineering course offered in fully face-to-face and hybrid course modes (*N* = 160). This course attempted to counteract trends of decreased student motivation and high attrition rates among engineering majors. Mixed-design analysis of variance examined differences in motivational constructs including student self-efficacy, effort regulation, and interest in engineering, as well as engineering skills throughout the course and across instructional modes. None of the motivational constructs were associated with significant decreases throughout the course nor with differences across instructional modes. However, students’ engineering skills increased throughout the course with no significant differences across course modalities. Furthermore, interest in engineering and effort regulation were positively associated with course performance. The instructional modality was not significantly associated with course performance. Overall, this study provides an example of a project-based introductory engineering course which may help maintain student motivation and foster student success in engineering.

## Introduction

Developing a strong science and engineering workforce is important for maintaining a strategic advantage in the global economy (National Academy of Sciences, National Academy of Engineering, & Institute of Medicine 2007). However, higher education institutions have had difficulty retaining students in engineering-related disciplines. Several studies have documented high attrition among engineering students (Geisinger and Raman 2013; Kokkelenberg and Sinha 2010; Rask 2010; Chen 2013). For example, one estimate shows that only 57% of engineering students stay in the major (Ohland et al. 2008). High attrition may stem in part from the type of curriculum students are exposed to early in their major. For instance, incoming engineering students experience frustration with theoretically focused curricula without any direct or meaningful connection to real-life applications (Dally and Zhang 1993). Research studies indicated that students choose to leave the engineering-related disciplines as they lose interest in engineering and become less motivated to pursue engineering careers, sometimes despite being in good academic standing (Besterfield-Sacre et al. 1997; Geisinger and Raman 2013).

Various constructs may influence motivation and persistence in pursuing engineering. For example, non-cognitive factors such as perception of engineering profession and self-confidence/efficacy are predictors of persistence (Burtner 2005). Similarly, Komarraju and Nadler (2013) examined the role of self-efficacy and effort regulation affecting motivation and academic achievement. Also, Alpay et al. (2008) examined student enthusiasm and found that students desire to make potential impact on society might help to maintain motivation throughout the program. Furthermore, Tendhar et al. (2017) reported a longitudinal study of students’ intention to persist in engineering career by examining expectancy for success and engineering identification.

In an effort to retain more engineering students in the major, institutions have revised pedagogical approach and developed first-year project-based curricula. The courses in project-based curricula are designed to provide application-oriented learning experiences that improve students’ self-efficacy and support the development of effort regulation (Klingbeil et al. 2004; Mills and Treagust 2003). These programs focus on teaching students engineering principles and concepts that can directly be applied to a practical design project, such as building assistive technology devices, quadcopters, or fitness-trackers (Carlson and Sullivan 1999). Studies have shown that these project-based learning experiences help students learn important engineering concepts and problem-solving skills (Pomalaza-Ráez and Groff 2003; Gavin 2011; Koch et al. 2017). Just as importantly, these courses have also been shown to improve motivation to persist in college engineering settings, in particular, if they emphasize the importance of hands-on, collaborative, and problem-based learning experiences (Knight et al. 2007; Pomalaza-Ráez and Groff 2003; Razzaq 2003).

This study is motivated to understand how project-based learning in an introductory engineering course can potentially help students persist in engineering majors (Bucks et al. 2015; Mills and Treagust 2003; Nguyen et al. 2020; Knight et al. 2007). Due to over-enrollment, we also developed a hybrid course (with online lectures and face-to-face laboratory sessions) that was identical to the fully face-to-face course. Consequently, this study examines how this project-based engineering course influences constructs related to major persistence, specifically on students’ self-efficacy, effort regulation, and interest in engineering.

## Conceptual framework

### Motivation, self-efficacy, effort regulation, and learning

One of the benefits of project-based learning is that the real-world nature of these experiences may help students understand important engineering concepts from both a theoretical and a practice nature. However, another important benefit of project-based learning is that they also increase student motivation towards learning. Achievement motivation, which is broadly viewed as the interest, energy, and engagement individuals put into self-directed learning behaviors (Wigfield et al. 2015). This type of motivation is often viewed as influential in determining student success and engagement during higher education and beyond. Achievement motivation constitutes a variety of different facets (see Wigfield and Eccles 2000 for a review) and has been shown to influence both success and persistence in engineering (French et al. 2005; Mamaril et al. 2016; Matusovich et al. 2010; Tendhar et al. 2018). Notably, student motivation to pursue an engineering career after graduation substantially decreases throughout the duration of undergraduate programs (Alpay et al. 2008; Jones et al. 2010). However, a recent longitudinal study that examined engineering students who persist through their first three years of their major indicated that students’ intention to pursue engineering careers did not significantly decrease in these three years (Tendhar et al. 2017). Nonetheless, constructs related to student motivation have been shown to correlate with learning and performance (e.g., Dweck 1986; Liu et al. 2012; Pintrich and De Groot 1990; Pinxten et al. 2019). Our study examines two important indicators of student motivation: self-efficacy and effort regulation.

*Self-efficacy* refers to students’ belief that they can perform well on a given task. In the context of higher education, numerous studies have shown self-efficacy to be an important predictor of success in engineering as measured by course performance and grade point average (e.g., Chyung et al. 2010; Loo and Choy 2013; Mamaril et al. 2016; Purzer 2011). However, studies examining engineering majors find large decreases in students’ self-efficacy over the course of their learning experiences. For instance, studies of incoming first-year students find that these students display significant decreases in their level of self-efficacy over the course of their studies (Jaeger and Adair 2018; Jones et al. 2010).

*Effort regulation* corresponds to students’ ability to persist in completing a learning task (Pintrich et al. 1993). What makes effort regulation different from other self-regulation strategies is that it considers self-regulation in the context of the more mundane and frustrating aspects of the learning experience. These include situations in which the learning activity is tedious, dull, or difficult, but is nonetheless necessary for achieving one’s learning goals. Effort regulation also captures the level of persistence students exhibit when trying to adhere to their learning schedule. Students who are said to have high effort regulation can complete a learning task, even if it is dull or boring, and are persistent about meeting their learning goals (Broadbent and Poon 2015). Studies have found that effort regulation is a positive predictor of academic achievement (Kim et al. 2015; Komarraju and Nadler 2013), and may therefore be important for understanding achievement in the context of engineering courses.

### The promise of project-based engineering courses

One useful pathway to increase student motivation and self-efficacy in engineering courses is to integrate hands-on, collaborative, and problem-based/project-based learning experiences (Jones et al. 2013; Nguyen et al. 2021). Characteristics of project-based learning include learning that is centered around an exemplary problem, focuses on activity-based experiences, is self-directed by students, and is group-based, among other characteristics (De Graaf and Kolmos 2003). Research on college-level engineering courses that had included such instructional characteristics, indicated gains in student motivation and learning, such as engagement in the course relative to other courses, and interest in engineering (Jones et al. 2013; Terrón-López et al. 2017; Yadav et al. 2011). Similarly, engineering courses that fostered self-regulated learning yielded increases in student motivation (Harding et al. 2007; Nelson et al. 2015). Additionally, engineering courses that emphasized collaboration and group learning experiences were shown to facilitate students’ self-efficacy (Hutchison et al. 2006; Hutchison-Green et al. 2008; Purzer 2011; Schaffer et al. 2012; Tlhoaele et al. 2016). Furthermore, project-based engineering courses can help develop students’ engineering skills by offering applied and practical learning experiences that foster student development of effective collaboration, communication, and coordination of multiple competencies, which Passow and Passows' (2017) systematic review identified as core engineering competencies. Not surprisingly, current engineering students and engineering college graduates have reported the high value of practical, hands-on experiences for both their personal and professional growth beyond college (Alpay et al. 2008; Chanson 2004; Gratchev and Jeng 2018).

### Understanding online and hybrid learning in higher education

Many engineering departments have embraced online learning in fully online and hybrid instructional modes because of their practical benefits to both students and departments (Bourne et al. 2005). For instance, online courses allow students to watch lectures and work on course material at their own convenience, providing students with greater flexibility in their course schedules (Waschull 2001). Also, one of the most practical benefits of online courses is that they can also help address issues with over-enrollment (Gould 2003; Lei and Gupta 2010). However, online course elements also pose some challenges to students. Research studies indicate that students often do not perform as well in online courses compared to students taking face-to-face courses (e.g., Fischer et al. 2020; Xu and Jaggars 2011, 2013, 2014). Some work has attributed these performance declines to students’ difficulty regulating their own learning process in online course environments. Because online courses do not meet regularly, students must have adequate self-efficacy and effort regulation skills to effectively manage their study time and keep track of important deadlines (Broadbent 2017; Parkes et al. 2015; You 2016). However, other work has noted that problems with learning online may also stem from the nature of online learning environments in general, in that they do not offer the same level of instructor engagement or peer interaction as face-to-face courses (Bernard et al. 2009; Jaggars and Xu 2016; Kuo et al. 2014). When fully online courses suffer from low student-instructor and peer interactions, students might feel less motivated in the course yielding worse learning outcomes.

### Research questions

This study aims to understand how of project-based engineering courses can contribute to important cognitive and non-cognitive learning outcomes for engineering education; namely self-efficacy, effort regulation, and interest in engineering, as well as engineering skills and overall course performance. While earlier research examined each individual construct, they have not been studied in tandem providing a unique opportunity of this study to contribute to the engineering education research base. Our research questions are as follows:

Research question 1: How does project-based learning affect student self-efficacy, effort regulation, and interest in engineering throughout the course?
Research question 2: How does project-based learning affect students’ perceived abilities in engineering skills throughout the course?
Research question 3: How is student self-efficacy, effort regulation, and interest in engineering associated with course performance?

## Methodology

### Study setting

This quantitative study examined a project-based engineering course, positioned for the first-year students in the curriculum, at a large public research university in the USA. This study was approved by the university’s human subjects review board. The study reports for two terms of study during the academic year of 2016–2017.

The course consisted of one-hour lectures and two-hour laboratory sessions on a weekly basis. This course occurred over two eleven-week terms, where the first half occurred in the Fall term and the second occurred in the Winter term. It was offered as a fully face-to-face course with face-to-face lecture and laboratory sessions, as well as a hybrid course with online lectures and online homework assignments but face-to-face laboratory sessions. Notably, the hybrid component was introduced to address over-enrollment and included 23 online video modules (average video time: 11.0 min) and 18 online video modules (average video time: 10.5 min) in the first and second term, respectively. The covered content was identical to the fully face-to-face course.

The main goals of this course were to (a) bridge the gap between analytical instruction and application of theory to an engineering project/problem and (b) train students to understand engineering design principles and develop problem-solving skills. This project-based engineering course emphasized engineering design, the integration of technical communications, and entrepreneurship. Since the course was not required, students from all engineering disciplines could enroll.

The main engineering project focused on designing, building, and testing a remote-controlled quadcopter. The technical lectures were on engineering topics relevant to quadcopter design. Furthermore, project management, product development, teamwork, and professional development were also integrated into the lecture component of the course. During laboratory sessions, engineering skills trainings (e.g., fabrication, computer-aided design, and programming) were provided, so that students could complete the course project by applying both practical skills from labs and theoretical understanding from lectures. During the second term, students continued their quadcopter project with an autonomous task with relevant technical contents. Both lectures and laboratory sessions were organized similar to the first term. However, the second term additionally focused on students’ professional development by having them create business plans related to their project. Students also attended professional talks by industry leaders who spoke about career options, current trends in research and technology, and offered advice about pursuing different engineering careers.

### Sample and data sources

Our sample consisted of students who enrolled in both terms of the two-term course sequence (first term, Fall 2016; second term, Winter 2017) and who did not switch course formats (face-to-face or hybrid) between terms (*N* = 160). There were 140 students in the face-to-face format and 20 students in the hybrid format. Notably, 19% of students dropped the course after the first term of the two-term sequence. Student surveys indicated that schedule conflicts were the main underlying reason. The majority of students in the sample were first-year students (84%) with a mean age of 18.4 years (standard deviation is 0.43 years). The sample was culturally diverse (35% Asian, 25% Latino, 21% Caucasian, 1.2% African American, and 17.8% others) and included substantially more male (81%) than female (19%) students. The incoming engineering students of 2016 consisted of 76% male and 24% female students, which the reported elective course attracted more male students. About 29% of students were classified as low-income students (i.e., based on family household income and household size using the 185% of the U.S. poverty line), about 40% of students were first-generation college students (i.e., neither parent hold a Bachelor’s degree), and 24% of students were English language learners. Data collected for this study included course-level data, institutional data, and web-based surveys. Course-level data included data on student course performance. Institutional data were obtained from the University Registrar and included student demographics and prior achievement. Web-based surveys were administered during the first and second terms of the course. The pre-survey was given in the first week of the first term (*T*_1_), the mid-survey was given at the end of the first term (*T*_2_), and the post-survey was given at the end of the second term of the course (*T*_3_).

This study restricted the analytical sample to students who agreed to participate in the study in exchange for course extra credit (3% extra credit that counted towards students’ final grade). These students participated in all three surveys (pre-, mid-, and post-surveys). For the face-to-face course, 140/190 students met these criteria, which accounted for 73.6% of the students. The hybrid course had 20/34 or 58.8% of students who met the criteria. Because our sample relied on whether students took all three surveys, we compared our final sample of 160 students to the sample of students who did not take the surveys. We found that our final sample had significantly higher final winter course grades (*M* = 11.49, *SD* = 1.30) than students who did not participate in the surveys (*M* = 11.06, *SD* = 1.08), *t*(222) = 2.55, *p* < 0.01.

### Measures

#### Course-level data

Course performance variables included final course grade for each of the two terms as a quasi-continuous variable (1 = F, 2 = D−, 3 = D, 4 = D+, …, 11 = A−, 12 = A, 13 = A+). The first term included three homework assignments and the second term included two homework assignments. Final course grade was based on a 60% team grade and a 40% individual grade. The individual grade consists of 10% on attendance, 10% on team evaluation, and 20% on homework. The team grade consists of 20% on design presentations, 20% on prototype structure and testing, and 20% on design report.

#### Institutional data

Institutional data included dichotomous variables describing students’ gender (0: Male, 1: Female), underrepresented minority status (0: Not an underrepresented minority, 1: underrepresented minority), low-income status (0: Not low-income, 1: Low-income), first-generation college student status (0: Not first-generation student, 1: First-generation student), and English language learning (ELL) status (0: Not ELL, 1: ELL). Furthermore, student college entrance examination scores were used as a continuous pre-college academic preparedness measure.

#### Web-based survey items

The *self-efficacy* scale was adopted from Midgley et al. (2000) and consisted of three 5-point Likert scale items (1 = Not at all true of me, 5 = Very true of me). These items read: (a) I will be able to master the skills taught in this course, (b) I'm certain I can figure out how to learn even the most difficult course material, and (c) I can do almost all the work in this class if I don't give up. Cronbach’s α of the self-efficacy scale was at 0.78, 0.82, and 0.83 for survey administration at T_1_, T_2_, and T_3_, respectively. Each item was averaged to create a composite score.

The *effort regulation* scale consisted of six 5-point Likert scale items (1 = Not at all true of me, 5 = Very true of me). Four of these items were taken from a widely used and validated effort regulation scale (Pintrich et al. 1993). These items read: (a) I often feel so lazy or bored when I study that I quit before I finish what I planned to do (reverse scored), (b) I work hard to do well in courses even if I don’t like what we are doing, (c) when coursework is difficult, I give up or only study the easy parts (reverse scored), and (d) even when course materials are dull and uninteresting, I manage to keep working until I finish. The remaining two items were developed by the authors of this study, which read: (e) When I make a schedule for my coursework, I stick to it, and (f) I am quick to get caught up with coursework if I start falling behind. Cronbach’s *α* of the effort regulation scale was at 0.62, 0.73, and 0.74 for survey administration at *T*_1_, *T*_2_, and *T*_3_, respectively. Each item was averaged to create a composite score.

Students’ *interest in engineering* was measured using continuous 10-point variables asking students to (a) rank their current interest in majoring in Engineering, and (b) to rank their current interest in pursuing a career in Engineering on scales of 1–10 (1 = Not interested at all and 10 = Extremely interested). These interest in engineering measures were developed by the university’s Engineering School assessment office and used for many years. While these items have not yet been strictly psychometrically validated, they nonetheless provided the Engineering School a straightforward way to understand students’ level of interest in past years.

Students’ *perceived engineering ability* variables consisted of student self-report on four continuous 5-point Likert scale-inspired variables (1 = Not confident/Don’t know, 5 = Very confident). These items were important to the context of the project-based engineering course and read: (a) ability to design and fabricate a device, (b) ability to use computer-aided design (CAD), (c) ability to implement the design process, and (d) ability to program a microcontroller or app.

### Analytical methods

The *first and second research questions* used 3 × 2 mixed-design analysis of variance (ANOVA) in order to assess how students’ self-rated motivation, interest in engineering, and engineering-related skills changed over time, and whether these scores differed between students in face-to-face and hybrid sections (Maxwell and Delaney 2004). In these 3 × 2 mixed-design ANOVA models, *time* was the within-subjects factor (*T*_1_ = pre-survey, *T*_2_ = mid-survey, *T*_3_ = post-survey) and *course format* was the between-subjects factor (face-to-face vs. hybrid). Assumptions of the mixed-design model were tested and did not violate assumptions (e.g., unequal variances, multicollinearity). For instance, for each measure in these mixed-design models (self-efficacy, effort regulation, interest in engineering, and engineering ability), Bartlett’s test was used to check for equality of variance assumptions. None of the models violated assumptions of equality of variance.

The *third research question* utilized the full data set to examine associations with student learning outcomes utilizing ordinary least square linear regression analysis (Montgomery et al. 2012). Model building was guided by both conceptual and statistical considerations to improve goodness-of-fit measures. Student course grades at the end of the second term served as the dependent variable. Independent variables include composite variables capturing self-efficacy and effort regulation, the single-item variable describing students’ interest in pursuing a career in engineering, as well as the dichotomous course modality variable (0: face-to-face course, 1: hybrid course). Covariates included student prior performance continuous variables (first term performance, entrance exam scores), and student demographic dichotomous variables (gender, racial/ethnical background, low-income status, first-generation college student status, English language learner status). Entrance exam scores, self-efficacy and effort regulation composite variables, and interest in engineering career variables were grand-mean centered and z-score transformed. Assumptions of the model were tested. For instance, multicollinearity was tested by calculating variance inflation factors (VIFs). Variables were removed from the model if VIFs were greater than three. An example of such a variable is students’ interest in majoring in engineering. Homoscedasticity was examined through residual versus fitted plots and the Breusch–Pagan test for heteroscedasticity. Consequently, the regression model used robust standard errors to account for heteroscedasticity in some variables. Missing data (below 1% for each variable) in the regression analysis was imputed with Markov Chain Monte Carlo multiple imputation methods with 150 iterations and 200 imputations (Cheema 2014; Graham 2009). Auxiliary variables (e.g., students’ engineering-related skills, interest in majoring in engineering) were used to improve the imputation model.

### Limitations

The most important limitations of this study relate to its data sources. As this study was situated at a selective research university, enrolled students in this introductory engineering course were well prepared for college. This might limit the extent to which inferences can be made for all engineering students globally. Also, since the course was not required for all engineering students, a selection bias may exist as students self-select into the course. Noteworthily, a prior study of the same course series that examined student motivation and interest in engineering did not find significant differences among students who chose to enroll in a prior iteration of this project-based engineering course and students who did not enroll in this course (Wu et al. 2016). However, students who completed all three surveys were more likely to have higher final course grades than students who did not complete the survey. Thus, it should be emphasized that the students in our sample represent the higher achieving students in the course. For replication studies, we encourage a randomized controlled trial study, which was not feasible at this institution, to reduce potential selection biases.

In addition, student ratings of self-efficacy, effort regulation, and interest in engineering were consistently high throughout the study. Despite prior validation of these scales (Midgley et al. 2000; Pintrich et al. 1993), our results may allude to a potential ceiling effect. In addition, engineering skills were measured using students’ self-reported ratings, which may not be objective.

The major threat to internal validity is that students participating in the two-term course sequence also enrolled in other college courses, which might have had influences on their self-efficacy, effort regulation, and interest in engineering. Furthermore, inferences from the hybrid and face-to-face comparisons need to be made with caution as potentially unobserved selection effects (e.g., prior experience with online courses, course schedules, etc.) might have occurred. Also, the low sample size of students in the hybrid course section is of concern. It resulted because of our inclusion criteria to only examine students enrolling in both terms in the same modality. As students were given freedom to switch between course modalities, some opted to enroll in the second term in a different course modality compared to the first term. Furthermore, 19% of the students enrolled in the first term did not continue the course during the second term mainly due to schedule conflicts. This low sample size of students in the hybrid course section substantially reduced the statistical power of the analysis. Power analysis utilizing G*Power 3.1 (Faul et al. 2009) indicate that this analysis is only able to detect effects with effect sizes above 0.168, which are considered medium-sized effects (Cohen 1992). Lastly, while students in the hybrid section were not allowed to attend face-to-face lectures, strict enforcement of this policy was limited due to logistic difficulties. Similarly, although students in the face-to-face sections of the course did not have access to the online lecture videos, they could technically have been exposed to the online lecture videos if they arranged lecture-viewings with friends attending the hybrid course section. However, anecdotal evidence from the course instructor indicates the absence of such course modality spillover effects.

## Results

### Project-based engineering course and student self-efficacy, effort regulation, and interest in engineering

**Fig. 1:**
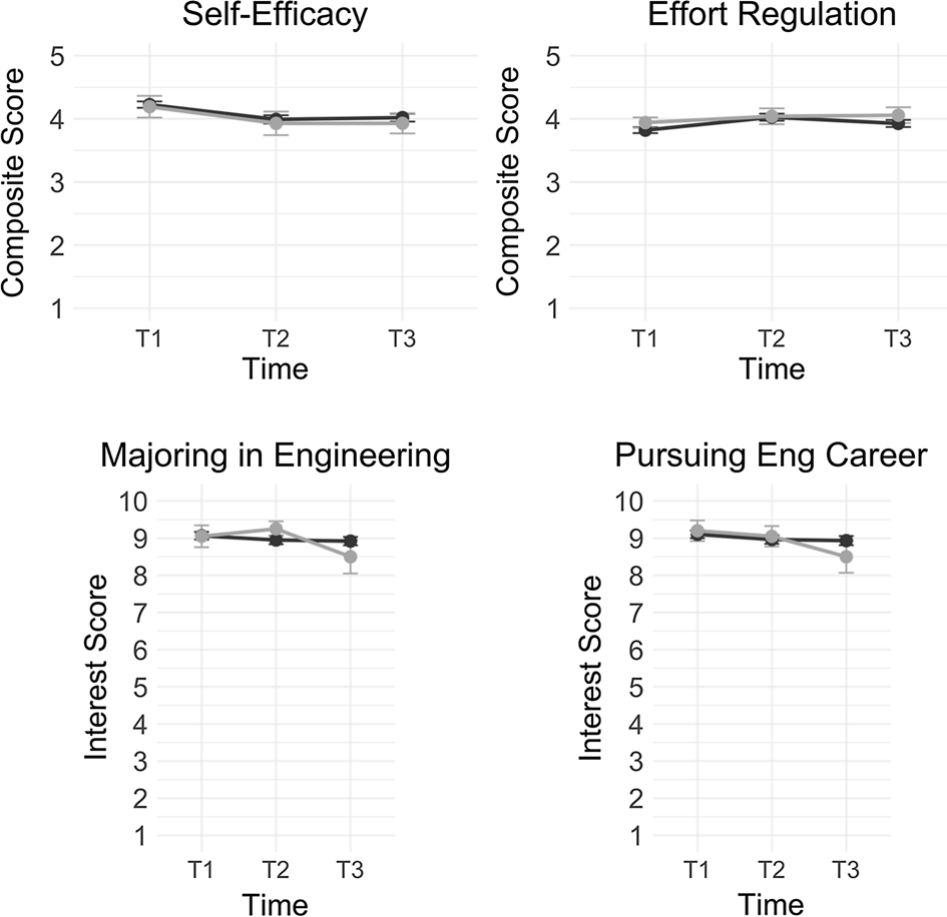
Comparisons of student self-efficacy, effort regulation, and interest in engineering across face-to-face (black lines) and hybrid course sections (grey lines)

The descriptive analysis indicated that all student perception variables (i.e., self-efficacy, effort regulation, interest in majoring in engineering, and interest in pursuing an engineering career) were at a high level at the beginning of the first term of the course (Fig. 1).

Mixed-design ANOVA were then applied to examine changes across the courses (Table 1). In particular, we did not detect significant changes in students’ self-efficacy throughout the course, *F*(2, 432) = 1.31, *p* = 0.256. Similarly, there were no significant changes in students’ effort regulation throughout the course, *F*(2, 438) = 0.54, *p* = 0.582. With respect to student interest in majoring in engineering, there were no significant changes throughout the course, *F*(2, 468) = 0.27, *p* = 0.760. Similarly, there were no significant changes in students’ interest in pursuing an engineering career throughout the course, *F*(2, 459) = 0.21, *p* = 0.805. Figure 1 illustrates these longitudinal trends throughout the two-term course.

**Table 1:** Descriptive statistics of student perceptions and engineering-related skills

		Overall						
		*N*	*T*_1_		*T*_2_		*T*_3_	
			Mean	SD	Mean	SD	Mean	SD
Perceptions	Self-efficacy^a^	147	4.22	0.60	3.98	0.75	4.01	0.71
	Effort regulation^a^	129	3.84	0.51	4.03	0.62	3.95	0.63
	Majoring in engineering^b^	159	9.06	1.24	8.99	1.30	8.87	1.45
	Pursuing engineering career^b^	156	9.12	1.21	8.97	1.33	8.88	1.52
Engineering-related skills	Fabricate device^a^	160	3.21	1.21	4.06	0.82	4.29	0.80
	Use CAD^a^	157	2.69	1.40	3.54	1.06	3.71	1.07
	Implement design process^a^	156	3.27	1.20	4.04	0.82	4.28	0.75
	Program a microcontroller^a^	158	2.61	1.20	–	–	3.37	1.10

		Face-to-face							Hybrid						
		*N*	*T*_1_		*T*_2_		*T*_3_		*N*	*T*_1_		*T*_2_		*T*_3_	
			Mean	SD	Mean	SD	Mean	SD		Mean	SD	Mean	SD	Mean	SD
Perceptions	Self-efficacy^a^	128	4.23	0.58	3.99	0.74	4.02	0.71	19	4.19	0.75	3.93	0.81	3.93	0.70
	Effort regulation^a^	129	3.82	0.53	4.03	0.64	3.93	0.64	20	3.94	0.37	4.04	0.56	4.06	0.56
	Majoring in engineering^b^	139	9.06	1.23	8.95	1.34	8.92	1.35	20	9.05	1.32	9.25	0.91	8.50	2.01
	Pursuing engineering career^b^	136	9.10	1.21	8.96	1.35	8.93	1.45	20	9.20	1.24	9.05	1.23	8.50	1.93
Engineering-related skills	Fabricate device^a^	140	3.18	1.99	4.05	0.80	4.27	4.40	20	3.35	1.26	4.05	0.88	4.40	0.68
	Use CAD^a^	137	2.73	1.38	3.51	1.06	3.67	3.95	20	2.35	1.42	3.75	1.01	3.95	0.82
	Implement design process^a^	136	3.26	1.20	4.01	0.83	4.26	0.76	20	3.30	1.26	4.25	0.71	4.35	0.67
	Program a microcontroller^a^	138	2.65	1.17	–	–	3.38	1.09	20	2.30	1.38	–	–	3.25	1.16

*CAD* computer-aided design, *T*_*1*_ pre-survey (beginning of first term), *T*_2_ end of first term survey, *T*_*3*_ end of second term survey

^a^5-point Likert scale item

^b^10-point scale item

Interestingly, there were no differences on these variables when comparing students in the face-to-face section to students in the hybrid section: self-efficacy (*F*(1, 432) = 0.40, *p* = 0.526), effort regulation (*F*(1, 438) = 1.28, *p* = 0.258), interest in majoring in engineering (*F*(1, 468) = 0.03, *p* = 0.800), and interest in pursuing an engineering career (*F*(1, 459) = 0.17, *p* = 0.675).

### Project-based engineering course and impact on student engineering skills

**Fig. 2:**
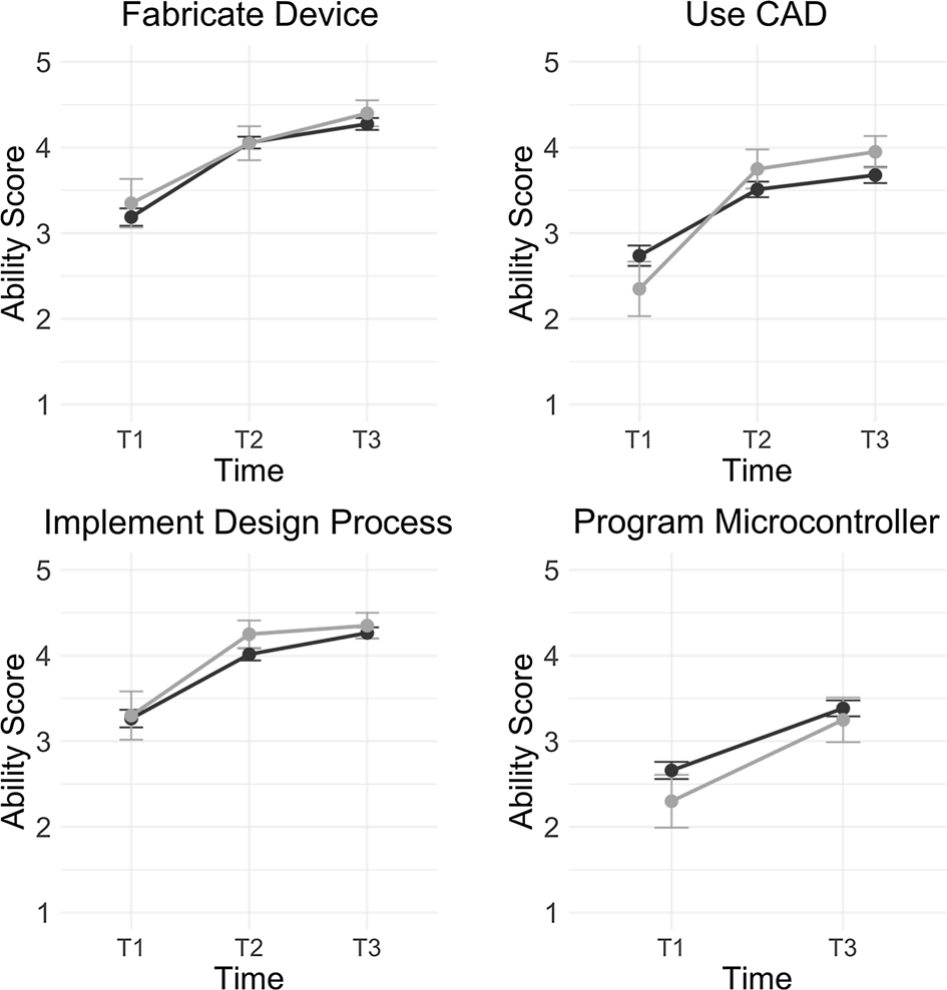
Comparisons of student engineering skills across face-to-face (black lines) and hybrid course sections (grey lines)

Descriptive analysis indicated that student self-reported engineering-related skills (i.e., fabricate a device, use CAD, implement a design process, and program a microcontroller) were low at Time 1 (pre-survey; Table 1). Figure 2 illustrates these trends throughout course.

Mixed-design ANOVA indicated significant increases in students’ ability to fabricate devices throughout the course, *F*(2, 465) = 24.33, *p* < 0.001. There was a significant increase in students’ self-reported ability to use CAD throughout the course, *F*(2, 462) = 10.58, *p* < 0.001. Students’ ability to implement design processes significantly increased throughout the course, *F*(2, 459) = 14.58, *p* < 0.001. Lastly, students’ ability to program a microcontroller significantly increased throughout the course, *F*(1, 310) = 12.83, *p* < 0.001. It is important to note that students did not learn how to program a microcontroller until the second term of the course. Because of this, we did not ask students about this skill at Time 2 (mid-survey).

Similar to the student perception variables, we did not find any significant differences across students in face-to-face and hybrid course sections: fabricate a device, (*F*(1, 465) = 0.37, *p* = 0.541), use CAD (*F*(1, 462) = 0.09, *p* < 0.756), implement design processes (*F*(1,459) = 0.69, *p* = 0.404), and program a microcontroller (*F*(1, 310) = 1.43, *p* = 0.231).

### Associations with course performance

Ordinary least squares linear regression analysis with robust standard errors identified several significant associations between motivational constructs and students’ final course grade at the end of the second term (*F*(11, 145.9) = 8.39, *p* < 0.001, *R*^2^ = 0.265; Table 2). Each standard deviation increase in students’ effort regulation was significantly associated with a 0.19 letter grade increase in their second term final course grade, *b* = 0.191, *t* = 2.07, *p* < 0.05. Additionally, each standard deviation increase in students’ interest in pursuing an engineering career was significantly associated with a 0.23 letter grade increase in their second term final course grade, *b* = 0.229, *t* = 1.99, *p* < 0.05. Notably, student self-efficacy was not significantly associated with student second term final course grade, *b* = -0.021, *t* =  − 0.25, *p* = 0.800*.* When comparing students by course modality, students enrolled in the hybrid course section were not found to have significantly different second term final course grades compared to students enrolled in the fully face-to-face section of this project-based introductory engineering course, *b* =  − 0.278, *t* =  − 1.19, *p* = 0.235. Finally, we also found that female students had an associated 0.49 letter grade increase when compared to male students, *b* = 0.492, *t* = 3.25, *p* < 0.01.

**Table 2:** Ordinary least squares linear regression with robust standard errors predicting final course grades at the end of the second term

	Coefficient	SE	*t*	*p*
Intercept	9.042	0.742	12.19	< 0.001
Hybrid course	− 0.278	0.233	− 1.19	0.235
Self-efficacy^a^	− 0.021	0.085	− 0.25	0.800
Effort regulation^a^	0.191	0.092	2.07	0.041
Pursuing engineering career^a^	0.229	0.115	1.99	0.049
End of first term course grade	0.223	0.061	3.66	< 0.001
Entrance exam scores^a^	0.069	0.076	0.90	0.368
Female	0.492	0.150	3.27	0.001
Underrepresented minority	− 0.289	0.202	− 1.43	0.155
Low-income	− 0.209	0.204	− 1.03	0.307
First-generation college student	0.160	0.171	0.94	0.351
English language learner	− 0.024	0.180	− 0.14	0.892

^a^Grand-mean centered and z-scored transformed

## Discussion

This study examined a two-part introductory project-based engineering course that occurred over a span of 22 weeks. This course design may allow us to better understand changes in motivational constructs beyond a single term of student enrollment. Consequently, this study examined associations among students’ self-efficacy, effort regulation, interest and skills in engineering, and performance across time and instructional modes. The main four findings of this study are as follows:

First, students who enrolled in the two-term course maintained high levels of self-efficacy, effort regulation, and interest in engineering. This is encouraging for students who opt to enroll in this project-based engineering course as student levels of interest in engineering typically decrease over the duration of engineering undergraduate programs including at our institution (e.g., Alpay et al. 2008; Jones et al. 2010). It is noted that the maintenance of interest in engineering in this study aligned with the longitudinal study conducted by Tendhar et al. (2017), in which students’ intention to pursue engineering careers did not significantly decrease in three years (from the first to the third years). Additionally, students’ high levels of self-efficacy and effort regulation provide a promising outlook as research emphasizes their importance for students’ short- and long-term success (e.g., Loo and Choy 2013; Mamaril et al. 2016). The course aimed at supporting student self-efficacy through hands-on engineering training, such as learning basic fabrication, soldering, and machining, as well as various project planning and technical presentations. Similarly, effort regulation was fostered through a series of project milestones. These findings may help illustrate how an introductory project-based engineering course can help retain student interest in engineering while providing students with opportunities to maintain and achieve high levels of self-efficacy and effort regulation.

Second, students’ self-reported engineering skills substantially increased throughout the course, and across course modalities. This corresponds to prior research indicating that project-based engineering courses foster student learning (e.g., Jones et al. 2013; Yadav et al. 2011). A similar study was conducted by Pomalaza-Ráez and Groff (2003) to increase student engineering competency specifically on computer programming skills in a robotic project. However, this study’s intention was to increase retention, and therefore did not quantitatively assess the increase in the computer programming. Our work was unique as we specifically observed increases in various students’ core engineering competencies, such as their ability to fabricate a device, use CAD to design a prototype, and program in a computer language—skills that are essential for working in industry (e.g., Alpay et al. 2008; Chanson 2004).

Third, motivational constructs—self-efficacy, effort regulation and interest in engineering, seem important for student course performance. In particular, effort regulation and interest in pursuing an engineering career were associated with higher end-of-course grades. These positive associations correspond to prior research that emphasizes the importance of effort regulation and interest for student learning (Harding et al. 2007; Liu et al. 2012; Nelson et al. 2015; Rodriguez et al. 2021). This is aligned to previous research studies that alluded to the importance of students' non-cognitive skills as indicators of success in engineering. For example, Lackey et al. (2003) found that engineering students' lab notebooks, in terms of organization and detail, was an important indicator of success in the class. Similarly, Zywno (2003) examined the important role of group work, and peer evaluations, in which these effort-based assignments could improve motivation and student learning outcomes. Furthermore, we found that female students had higher final course grades than male students. This is notable as prior research has emphasized the importance of providing learning experiences that can help not only attract female students to the discipline but also retain them (Du and Kolmos 2009; Kolmos et al. 2013). Our results suggest that some improvements at the curricular-level, namely project-based and team-based learning, may provide female students with a positive learning experience and also potentially contribute to their early success in engineering majors.

Fourth, the hybrid course section did not seem to provide different educational experiences compared to the fully face-to-face course section. Students in the hybrid section who learned the technical topics through online video modules exhibited similar trends in all measured engineering skills and did not have negative declines in self-efficacy, effort regulation, interest in engineering, or course grades. Nonetheless, this study provides initial support to integrate hybrid instruction in introductory engineering courses without substantially degrading educational experiences. This is of importance when considering that departments may want to retain some distance learning elements after the COVID-19 induced shift to emergency distance education.

## Conclusion

Overall, this study provides an encouraging example of how a project-based introductory engineering course might counteract typical trends of reduced motivation, self-efficacy, and interest in engineering. This type of project-based and team-based course modality especially provides a positive impact on female students for better academic performances in engineering. Although the study was conducted in a U.S. institution, educators globally valued the importance of promoting students’ persistence and motivation in engineering (Koch et al. 2017). The encouraging findings of this study indicate that a potential avenue to address this need may be found in the implementations of project-based engineering courses. Furthermore, this study is relevant to the current teaching modality during the pandemic. Although the study setting of incorporating hybrid course formats was pre-pandemic, the results indicated hybrid modalities may be as effective as in-person courses, which may help departments see value in online course offerings even after the pandemic (Bourne et al. 2005; Fischer et al. 2019; Lei and Gupta 2010). Future studies might further examine the role of project-based, hands-on introductory engineering courses and the development of self-efficacy, effort regulation and interest in engineering on more distal student success factors such as major persistence, retention, graduation rates, and time-to-degree. Furthermore, replication studies at institutions of higher education across the globe with different student populations are encouraged to increase the generalizability of the results, ultimately attempting to better support engineering students throughout their college career.

## Data Availability

The datasets generated during and/or analyzed during the current study are not publicly available due the IRB agreement and student privacy protection regulations. However, an anonymized data set could be made available from the corresponding author on reasonable request.

## References

[CR2] Alpay E, Ahearn AL, Graham RH, Bull AMJ (2008). Student enthusiasm for engineering: charting changes in student aspirations and motivation. Eur J Eng Educ.

[CR3] Bernard RM, Abrami PC, Borokhovski E, Wade CA, Tamim RM, Surkes MA, Bethel EC (2009). A meta-analysis of three types of interaction treatments in distance education. Rev Educ Res.

[CR4] Besterfield-Sacre M, Atman CJ, Shuman LJ (1997). Characteristics of freshman engineering students: models for determining student attrition in engineering. J Eng Educ.

[CR5] Bourne J, Harris D, Mayadas F (2005). Online engineering education: learning anywhere, anytime. J Eng Educ.

[CR6] Broadbent J (2017). Comparing online and blended learner’s self-regulated learning strategies and academic performance. Internet High Educ.

[CR7] Broadbent J, Poon WL (2015). Self-regulated learning strategies & academic achievement in online higher education learning environments: a systematic review. Internet High Educ.

[CR8] Bucks GW, Ossman KA, Kastner J, Boerio FJ (2015) First-year engineering courses: effect on retention and workplace performance. Paper presented at the 2015 American Society of Engineering Education Annual Conference, Seattle, WA

[CR9] Burtner J (2005). The use of discriminant analysis to investigate the influence of non-cognitive factors on engineering school persistence. J Eng Educ.

[CR10] Carlson LE, Sullivan JF (1999). Hands-on engineering: learning by doing in the integrated teaching and learning program. Int J Eng Educ.

[CR11] Chanson H (2004). Enhancing students’ motivation in the undergraduate teaching of hydraulic engineering: role of field works. J Prof Issues Eng Educ Pract.

[CR12] Cheema JR (2014). A review of missing data handling methods in education research. Rev Educ Res.

[CR13] Chen X (2013) STEM attrition: college students’ paths into and out of STEM fields (Statistical analysis report NCES 2014-001). National Center for Education Statistics, Institute of Education Sciences, U.S. Department of Education

[CR14] Chyung SY, Moll AJ, Berg SA (2010). The role of intrinsic goal orientation, self-efficacy, and e-learning practice in engineering education. J Effect Teach.

[CR15] Cohen J (1992). A power primer. Psychol Bull.

[CR16] Dally JW, Zhang GM (1993). A freshman engineering design course. J Eng Educ.

[CR17] De Graaf E, Kolmos A (2003). Characteristics of problem-based learning. Int J Eng Educ.

[CR18] Du X, Kolmos A (2009). Increasing the diversity of engineering education–a gender analysis in a PBL context. Eur J Eng Educ.

[CR19] Dweck CS (1986). Motivational processes affecting learning. Am Psychol.

[CR20] Faul F, Erdfelder E, Buchner A, Lang A-G (2009). Statistical power analyses using G*Power 3.1: tests for correlation and regression analyses. Behav Res Methods.

[CR21] Fischer C, Zhou N, Rodriguez F, Warschauer M, King S (2019). Improving college student success in organic chemistry: impact of an online preparatory course. J Chem Educ.

[CR22] Fischer C, Xu D, Rodriguez F, Denaro K, Warschauer M (2020). Effects of course modality in summer session: enrollment patterns and student performance in face-to-face and online classes. Internet High Educ.

[CR23] French BF, Immekus JC, Oakes WC (2005). An examination of indicators of engineering students’ success and persistence. J Eng Educ.

[CR24] Gavin K (2011). Case study of a project-based learning course in civil engineering design. Eur J Eng Educ.

[CR25] Geisinger BN, Raman DR (2013). Why they leave: understanding student attrition from engineering majors. Int J Eng Educ.

[CR26] Gould T (2003) Hybrid classes: maximizing institutional resources and student learning. In: Proceedings of the 2003 ASCUE conference

[CR27] Graham JW (2009). Missing data analysis: making it work in the real world. Annu Rev Psychol.

[CR103] Gratchev I, Jeng DS (2018). Introducing a project-based assignment in a traditionally taught engineering course. Eur J Eng Educ.

[CR28] Harding TS, Vanasupa L, Savage RN, Stolk JD (2007) Work-in-progress-self-directed learning and motivation in a project-based learning environment. In: Frontiers in education conference-global engineering: knowledge without borders, opportunities without passports, 2007. FIE’07. 37th Annual, F2G-3

[CR29] Hutchison MA, Follman DK, Sumpter M, Bodner GM (2006). Factors influencing the self-efficacy beliefs of first-year engineering students. J Eng Educ.

[CR30] Hutchison-Green MA, Follman DK, Bodner GM (2008). Providing a voice: qualitative investigation of the impact of a first-year engineering experience on students’ efficacy beliefs. J Eng Educ.

[CR100] Jaeger M, Adair D (2018). Transitioning from diploma to degree–impact on engineering students’ self-efficacy, expectancies, values and self-regulation. Eur J Eng Educ.

[CR31] Jaggars SS, Xu D (2016). How do online course design features influence student performance?. Comput Educ.

[CR32] Jones BD, Paretti MC, Hein SF, Knott TW (2010). An analysis of motivation constructs with first-year engineering students: relationships among expectancies, values, achievement, and career plans. J Eng Educ.

[CR33] Jones BD, Epler CM, Mokri P, Bryant LH, Paretti MC (2013). The effects of a collaborative problem-based learning experience on students’ motivation in engineering capstone courses. Interdiscip J Probl-Based Learn.

[CR34] Kim C, Park SW, Cozart J, Lee H (2015). From motivation to engagement: the role of effort regulation of virtual high school students in mathematics courses. Educ Technol Soc.

[CR35] Klingbeil N, Mercer RE, Rattan KS, Raymer ML, Reynolds DB (2004) Rethinking engineering mathematics education: a model for increased retention, motivation, and success in engineering. In: Proceedings of the 2004 American Society for engineering education annual conference and exposition. ASEE 2004, Salt Lake City, UT

[CR36] Knight D, Carlson LE, Sullivan JF (2007) Improving engineering student retention through hands-on, team based, first-year design projects, 13

[CR37] Koch FD, Dirsch-Weigand A, Awolin M, Pinkelman RJ, Hampe MJ (2017). Motivating first-year university students by interdisciplinary study projects. Eur J Eng Educ.

[CR38] Kokkelenberg EC, Sinha E (2010). Who succeeds in STEM studies? An analysis of Binghamton University undergraduate students. Econ Educ Rev.

[CR39] Kolmos A, Mejlgaard N, Haase S, Holgaard JE (2013). Motivational factors, gender and engineering education. Eur J Eng Educ.

[CR40] Komarraju M, Nadler D (2013). Self-efficacy and academic achievement: why do implicit beliefs, goals, and effort regulation matter?. Learn Individ Differ.

[CR41] Kuo Y-C, Walker AE, Schroder KEE, Belland BR (2014). Interaction, internet self-efficacy, and self-regulated learning as predictors of student satisfaction in online education courses. Internet High Educ.

[CR42] Lackey LW, Lackey WJ, Grady HM, Davis MT (2003). Efficacy of using a single, non-technical variable to predict the academic success of freshmen engineering students. J Eng Educ.

[CR43] Lei SA, Gupta RK (2010). College distance education courses: evaluating benefits and costs from institutional, faculty and students’ perspectives. Education.

[CR44] Liu OL, Bridgeman B, Adler RM (2012). Measuring learning outcomes in higher education: motivation matters. Educ Res.

[CR45] Loo CW, Choy JLF (2013). Sources of self-efficacy influencing academic performance of engineering students. Am J Educ Res.

[CR46] Mamaril NA, Usher EL, Li CR, Economy DR, Kennedy MS (2016). Measuring undergraduate students’ engineering self-efficacy: a validation study: measuring undergraduate students’ engineering self-efficacy. J Eng Educ.

[CR47] Matusovich HM, Streveler R, Miller R (2010). Why do students choose engineering? A qualitative, longitudinal investigation of students’ motivational values. J Eng Educ.

[CR48] Maxwell SE, Delaney HD (2004). Designing experiments and analyzing data: a model comparison perspective.

[CR49] Midgley C, Maehr ML, Hruda LZ, Anderman E, Anderman L, Freeman KE, Gheen M, Kaplan A, Kumar R, Middleton MJ, Nelson J, Roeser R, Urdan T (2000). Patterns of adaptive learning scales.

[CR50] Mills JE, Treagust DF (2003). Engineering education—is problem-based or project-based learning the answer?. Australas J Eng Educ.

[CR51] Montgomery DC, Peck EA, Vining GG (2012). Introduction to linear regression analysis.

[CR52] National Academy of Sciences, National Academy of Engineering, & Institute of Medicine (2007) Rising above the gathering storm: energizing and employing America for a brighter economic future. National Academies Press

[CR53] Nelson KG, Shell DF, Husman J, Fishman EJ, Soh L-K (2015). Motivational and self-regulated learning profiles of students taking a foundational engineering course: learning profiles of students in a foundational rngineering course. J Eng Educ.

[CR54] Nguyen H, Wu L, Fischer C, Washington G, Warschauer M (2020). Increasing success in college: examining the impact of a project-based introductory engineering course. J Eng Educ.

[CR55] Nguyen H, Lim KY, Wu LL, Fischer C, Warschauer M (2021). “We’re looking good”: Social exchange and regulation temporality in collaborative design. Learn Instr.

[CR56] Ohland MW, Sheppard SD, Lichtenstein G, Eris O, Chachra D, Layton RA (2008). Persistence, engagement, and migration in engineering programs. J Eng Educ.

[CR57] Parkes M, Stein S, Reading C (2015). Student preparedness for university e-learning environments. Internet High Educ.

[CR58] Passow HJ, Passow CH (2017). What competencies should undergraduate engineering programs emphasize? A systematic review. J Eng Educ.

[CR59] Pintrich PR, De Groot EV (1990). Motivational and self-regulated learning components of classroom academic performance. J Educ Psychol.

[CR60] Pintrich PR, Smith DAF, Garcia T, McKeachie WJ (1993). Reliability and predictive validity of the motivated strategies for learning questionnaire (MSLQ). Educ Psychol Measur.

[CR61] Pinxten M, Van Soom C, Peeters C, De Laet T, Langie G (2019). At-risk at the gate: prediction of study success of first-year science and engineering students in an open-admission university in Flanders—any incremental validity of study strategies?. Eur J Psychol Educ.

[CR62] Pomalaza-Ráez C, Groff BH (2003). Retention 101: where robots go … students follow. J Eng Educ.

[CR63] Purzer S (2011). The relationship between team discourse, self-efficacy, and individual achievement: a sequential mixed-methods study. J Eng Educ.

[CR64] Rask K (2010). Attrition in STEM fields at a liberal arts college: the importance of grades and pre-collegiate preferences. Econ Educ Rev.

[CR65] Razzaq Z (2003) An effective teaching strategy for motivation and retention of engineering and technology freshmen. In: Proceedings of the 2003 American Society for engineering education annual conference

[CR66] Rodriguez F, Fischer C, Zhou N, Warschauer M, Massimelli Sewall J (2021). Student spacing and self-testing strategies and their associations with learning in an upper division microbiology course. SN Soc Sci.

[CR67] Schaffer SP, Chen X, Zhu X, Oakes WC (2012). Self-efficacy for cross-disciplinary learning in project-based teams. J Eng Educ.

[CR70] Tendhar C, Paretti MC, Jones BD (2017). The effects of gender, engineering identification, and engineering program expectancy on engineering career intentions: applying hierarchical linear modeling (HLM) In engineering education research. Am J Eng Educ.

[CR71] Tendhar C, Singh K, Jones BD (2018). Using the domain identification model to study major and career decision-making processes. Eur J Eng Educ.

[CR101] Terrón-López MJ, García-García MJ, Velasco-Quintana PJ, Ocampo J, Vigil Montaño MR, Gaya-López MC (2017). Implementation of a project-based engineering school: increasing student motivation and relevant learning. Eur J Eng Educ.

[CR102] Tlhoaele M, Suhre C, Hofman A (2016). sing technology-enhanced, cooperative, group-project learning for student comprehension and academic performance. Eur J Eng Educ.

[CR72] Waschull SB (2001). The online delivery of psychology courses: attrition, performance, and evaluation. Teach Psychol.

[CR73] Wigfield A, Eccles JS (2000). Expectancy–value theory of achievement motivation. Contemp Educ Psychol.

[CR74] Wigfield A, Eccles JS, Fredricks JA, Simpkins S, Roeser RW, Schiefele U (2015) Development of achievement motivation and engagement. In: Handbook of child psychology and developmental science, pp 1–44

[CR75] Wu LL, Cassidy RM, McCarthy JM, LaRue JC, Washington GN (2016) Implementation and impact of a first-year project-based learning course. In: 2016 ASEE annual conference & exposition, New Orleans, LA

[CR76] Xu D, Jaggars SS (2011). The effectiveness of distance education across Virginia’s community colleges: evidence from introductory college-level Math and English courses. Educ Eval Policy Anal.

[CR77] Xu D, Jaggars SS (2013). The impact of online learning on students’ course outcomes: evidence from a large community and technical college system. Econ Educ Rev.

[CR78] Xu D, Jaggars SS (2014). Performance gaps between online and face-to-face courses: differences across types of students and academic subject areas. J High Educ.

[CR79] Yadav A, Subedi D, Lundenberg MA, Bunting CF (2011). Problem-based learning: influence on students’ learning in an electrical engineering course. J Eng Educ.

[CR80] You JW (2016). Identifying significant indicators using LMS data to predict course achievement in online learning. Internet High Educ.

[CR81] Zywno MS (2003). Using collaborative learning and peer assessment in an undergraduate engineering course: a case study. World Trans Eng Technol Educ.

